# Characterizing Origin of Differences in Chemical Composition, Rumen Degradability, Intestinal Digestibility and Amino Acid Profile of Traditional Beer Brewing Byproducts

**DOI:** 10.1002/fsn3.71380

**Published:** 2025-12-30

**Authors:** Alemayehu Tadesse, Nympha De Neve, Yayneshet Tesfay, Pieter Vermeir, Bruno De Meulenaer, Veerle Fievez

**Affiliations:** ^1^ Department of Animal, Rangeland and Wildlife Sciences Mekelle University Mekelle Ethiopia; ^2^ Department of Animal Sciences and Aquatic Ecology, Faculty of Bioscience Engineering Ghent University Ghent Belgium; ^3^ Independent Consultant Mekelle Ethiopia; ^4^ Laboratory for Chemical Analysis (LCA), Department of Green Chemistry and Technology, Faculty of Bioscience Engineering Ghent University Ghent Belgium; ^5^ Department of Food Technology, Food Safety and Health, Faculty of Bioscience Engineering Ghent University Ghent Belgium

**Keywords:** amino acid, atella, cereal type, heat treatment, in vitro, nylon bag

## Abstract

In tropical regions, protein is a critically limiting nutrient for dairy cow milk production, prompting the exploration of alternative protein sources. Brewery byproducts have been recognized for their wide use as valuable protein sources in ruminant nutrition. Traditional fermented beers are widely produced and consumed in Asia and Africa. This study evaluated the effects of heat treatment and cereal type to produce tella (Ethiopian traditional household beer) on crude protein (CP) and amino acid (AA) rumen degradability and intestinal digestibility of atella (a byproduct of tella). This was assessed by an in vitro simulation of the digestive process. Tella was prepared using a 0.25:0.03:0.02:0.70 ratio of maize or sorghum flour, barley malt, local hops, and water. Two tella types were produced: low‐temperature‐treated (109°C ± 9.4°C for 19 ± 4.8 min) and high‐temperature‐treated (159°C ± 16.1°C for 17 ± 2.9 min). Atella samples were incubated in an in vitro rumen simulation for 2, 4, 6, 10, 24, 48 and 72 h to assess dry matter (DM) and CP degradability. Intestinal digestibility was determined by exposure of the 10‐h rumen residues to pepsin and pancreatic enzymes. Cereal type‐dependent variations were observed (*p* < 0.05) in rumen degradation of DM, CP and AA, except for tryptophan. Heat treatment did not influence the ruminal degradation of DM, CP and AA, except for valine. Intestinal digestibility of bypass CP and all AA was affected (*p* < 0.05) by cereal type or heat treatment. Low‐temperature‐treated maize‐derived atella showed higher total in vitro CP digestibility and intestinal digestibility of most AA compared to other atella types. Lysine and methionine were identified as primary and secondary limiting amino acids for milk protein production. Given the relatively high proportions of rumen undegradable protein in cereal‐based atella diets, they should be balanced with feed ingredients high in rumen degradable protein.

## Graphical abstract

This study evaluated the effects of heat treatment and cereal type to produce tella (a traditional Ethiopian household beer) on crude protein (CP) and amino acid (AA) rumen degradability and intestinal digestibility of traditional beer brewing byproduct (atella). Temperature differences in preparation of tella didn't affect rumen CP/AA degradation. Low‐temperature‐treated atella had better intestinal digestibility of CP and AA. Cereal type influenced CP/AA rumen degradation and post‐ruminal digestibility. Maize‐derived atella had more rumen undegradable and intestinally digestible protein.

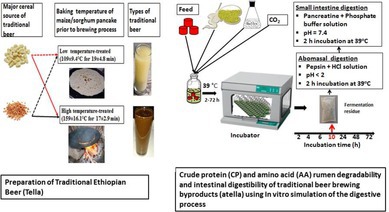

## 1 Introduction

Beer is the second most consumed alcoholic beverage worldwide, accounting for 34.3% of global alcohol consumption (WHO 2018). Traditional fermented beers are widely produced and consumed across Asia (Lee and Kim 2016) and Africa (Fentie et al. 2020; Sawadogo‐Lingani et al. 2021). The byproducts generated from these traditional brewing processes play a significant role in the diets of ruminants in these regions, serving as a crucial nutritional source.

Tella, a traditional Ethiopian beer, is brewed at household level (Tadesse and Tesfay 2011) from various cereals including maize ( *Zea mays* L.), sorghum [ *Sorghum bicolor* (L.) *Moench*], barley ( *Hordeum vulgare* ), wheat ( *Triticum aestivum* L.), and finger millet ( *Eleusine coracana* ). The choice of these ingredients depends on availability, price, and individual household preferences. Fermentation procedures of tella largely follow the principles of the industrial beer brewery but are preceded by baking of a cereal pancake. Differences in temperature and duration of this process mainly determine variation in physical properties of tella such as color and turbidity, with brown and light‐yellow colored tella originating from a high‐ and low‐temperature dry heat treatment, respectively.

Atella is the wet (80%–86% moisture) fermentation residue of tella (Tadesse and Tesfay 2011). It is a year‐round available and cheap supplement for dairy cattle in Ethiopia (Tadesse et al. 2022), with a crude protein (CP) content ranging between 16.5% and 24% on a DM basis (Demeke 2007; Tadesse and Tesfay 2011; Tadesse et al. 2022). This variation is attributed to differences in ingredients and their proportion (Tadesse and Tesfay 2011). The large proportion of bypass protein (70%) is a challenging feature of the crude protein of atella in which degradable protein may not be sufficient to meet the rumen microbial needs (Tadesse et al. 2022). Thus, the bypass protein needs to have high post‐ruminal digestibility and a favorable digestible amino acids (AA) composition to complement microbial protein from the rumen (Vaga et al. 2017).

The high resistance against microbial degradation of atella protein may be related to the grain's heat treatment before the brewing process. Indeed, a reduction in rumen protein degradation has been reported upon heat treatment of whole cotton seed (Arieli et al. 1989), soybean seed (Ganesh and Grieve 1990), canola meal (McKinnon et al. 1995), mustard meal (Mustafa et al. 1999), and hempseed cake (Karlsson et al. 2012). The magnitude of response in the reduction of rumen degradable protein (RDP) depends on the heating method, such as the temperature (McKinnon et al. 1995; Dakowski et al. 1996), the duration (Arieli et al. 1989; McKinnon et al. 1995; Vaga et al. 2017), and the form of heat treatment (dry vs. wet; Vaga et al. 2017). Further, the AA composition of the protein plays a role. Among the essential AA, lysine seems more susceptible to heat treatment as suggested from results of canola meal (Moshtaghi Nia and Ingalls 1995) and corn (Costa et al. 1977). This was confirmed in a study by Dakowski et al. (1996) with rapeseed meal exposed to increasing heating temperature from 130°C to 150°C which resulted in a reduction in the intestinal digestibility of individual amino acids in the rumen undegraded residue. Decreases ranged from 6.6% to 16.6%, with lysine and methionine specifically showing a decrease of 14.6% and 15.1%, respectively.

Despite the wide use of atella in the dairy ration across Ethiopia, little is known about its nutritive characteristics and their relation with the cereal type (mostly used are maize and sorghum) and the baking temperature (high vs. low) of the pancake. This study advances feedstuff characterization by moving beyond traditional proximate analysis and in vitro rumen crude protein degradation (Tadesse et al. 2022) to provide a fully integrated assessment of protein and amino acid availability. By simultaneously evaluating ruminal degradation and small intestinal digestibility at both the protein and individual amino acid levels, this study offers a more precise understanding of nutrient utilization. Crucially, it does so in relation to the origin of the byproduct—considering both the grain source (maize vs. sorghum) and the processing conditions (high‐ vs. low‐heat treatments)—thereby enabling a more refined characterization of how intrinsic protein structures of feedstuff and processing interact to influence protein utilization. These novel aspects contribute to the pool of knowledge necessary for optimized diets required for improved ruminant nutrition.

Accordingly, this in vitro study was conducted to determine the effect of heat treatment as well as the type of cereal to produce tella on CP and AA rumen degradability and intestinal digestibility of atella.

## 2 Materials and Methods

### 2.1 Preparation of Tella and Sampling of Atella

A total of 30 tella makers were randomly selected from Mekelle city and interviewed about the production process of the commonly produced tella types. Based on this survey result, the present study was designed emphasizing the preparation of the two types of tella [brown‐colored (high‐temperature heat treatment) and light yellow‐colored (low‐temperature heat treatment; see further for details)]. From the interviewed tella makers, four of them were randomly selected to prepare 10 L brown and light yellow colored tella from maize ( *Zea mays* ) and sorghum ( *Sorghum bicolor* ), with ingredients' proportions of baked maize or sorghum flour, barley malt, local hops (gesho), and water of 0.25:0.03:0.02:0.70.

Brown‐colored tella was prepared from maize or sorghum flour that was baked on a steel plate (⌀ 60 cm) kept on a three‐stone wood fire at 159°C ± 16.1°C for 17 ± 2.9 min. While in the light‐yellow‐colored tella, maize or sorghum flour dough was baked in a conventional electric injera mitad (stove) fitted with a clay plate (⌀ 58 cm) at 109°C ± 9.4°C for 19 ± 4.8 min. Afterwards, both kita (pancakes) were treated in the same way to produce tella. The detailed production process of tella preparation consisted of three fermentation phases: (1) *Primary fermentation*: Grounded barley malt and half of the quantity of the local hops powder were mixed with water and fermented for 3 days, resulting in a product known as “tinsis” (2) *Secondary fermentation*: The baked pancakes (kita) of maize or sorghum were broken into small pieces which were mixed along with tinsis, the other half of the quantity of the local hops powder and water and then fermented for 3 days. The product of this secondary fermentation is known as “difdif” (3) *Tertiary fermentation*: In this phase, difdif was mixed with water and then fermented for 2 days, the final product of the tertiary fermentation (tella) was harvested by filtration on the muslin cloth; subsequently, the wet fermentation residue remaining on the muslin cloth is named as “atella”.

The atella samples which were recovered from different types of tella from the four tella makers were collected separately in a 2 L plastic container. The atella samples which initially had a high moisture content were dried in an air oven at 50°C for 48 h. Atella samples were ground and sieved. Chemical and in vitro experiment analyses were performed on particles passing a 1 mm sieve, but maintained on the 0.2 mm sieve.

### 2.2 Chemical Analysis and Other Lab Measurements

The dry matter (DM) analyses were done by drying samples in a forced air oven at 103°C for 4 h and the nitrogen was determined using the micro Kjeldahl method and converted to CP using the factor 6.25 (AOAC 1990). Dry material was used to determine the degradation of DM, CP and AA profile. The original atella sample, along with residues from the 10‐h rumen incubation and the pepsin/pancreatin digestion simulation, underwent AA profile analysis. Acid hydrolysis was conducted in 6 M HCl in sealed tubes at 105°C for 24 h (excluding methionine, cysteine, and tryptophan). Tryptophan hydrolysis was performed in an alkaline solution using 4.2 M NaOH at 105°C for 20 h, following the Commission Directives 98/64/EC (European Commission 1998). The extraction and quantification of methionine and cysteine using reference material were inaccurate and imprecise due to technical issues, necessitating the indirect estimation of their levels based on sulfur content (see further). The hydrolysates were derivatized with *o*‐phthalaldehyde (OPA) or 9‐fluorenylmethyl chloroformate (FMOC) solutions as described by Kerkaert et al. (2011). Amino acids were then separated using a High‐Performance Liquid Chromatograph (Agilent Infinity 1260 with AdvanceBio Amino Acid Analysis (AAA), 3.0 × 100 mm, LC column from Agilent with guard column of the same type) system and detected with a fluorescence detector as outlined by Schuster (1988). The sulfur content in the original atella sample, as well as in residues following a 10‐h rumen incubation and subsequent pepsin‐pancreatin digestion, was determined following acid digestion in the microwave and inductive coupled plasma optical emission spectroscopy (ICP‐OES) analysis. Approximately 0.25 g of each sample was weighed into microwave digestion tubes, to which 5 mL of HNO_3_ were added and the samples were digested using a microwave (Milestone Ultrawave). The digestates were quantitatively transferred to 50 mL flasks and diluted to the mark with milliQ‐water. These solutions were filtered over a whatman 5 filter into test tubes and were quantified using ICP‐OES (iCAP 7000, thermos Fisher) according to the ISO 11885. As indicated before, sulfur‐containing amino acids (methionine and cysteine) were quantified based on the sulfur content of the sample. Based on literature values from the Feedipedia Animal Feed Resources Information System (Heuzé et al. 2015, 2017), we estimated the typical levels of methionine and cysteine in maize (1.7 and 2.1 g/100 g CP, respectively) and sorghum (1.9 and 1.9 g/100 g CP, respectively). From these levels, we calculated the ratio of cysteine to methionine (60.3/39.7 for maize and 44.8/55.2 for sorghum, respectively). Using these proportions in combination with the molar proportion of sulfur in methionine (21.5%) and cysteine (26.4%), the average proportion of sulfur in the sulfur‐containing amino acids was calculated to represent 24.45% for maize and 24.11% for sorghum. The acid detergent fiber (ADF) was determined using the ANKOM^200^ fiber analyzer (ANKOM technology, Macedon, NY, USA) following the procedure elaborated in Van Soest et al. (1991). The acid detergent insoluble CP was determined by Kjeldahl analysis of the ADF residues.

Color coordinates (CIE L*a*b*) were measured with a Hunter Lab Miniscan Minolta XE plus spectrocolorimeter (USA). The L*, a*, and b* values are a measure of lightness, redness, and yellowness, respectively.

### 2.3 In Vitro Incubation

Rumen degradability of DM and CP samples was determined using the in vitro procedure elaborated by Lima‐Orozco et al. (2014). Atella samples weighing 250 ± 1 mg (500 ± 1 mg in the 10 h series) were sealed in nylon bags (Solana, Edegem, Belgium, pore size 37 μm) and incubated in a shaking incubator at 39°C in flasks containing 25 mL (50 mL for the 10 h series flasks) of a buffer/rumen fluid mixture under CO_2_ environment. The buffer was prepared according to the procedure described by Lima‐Orozco et al. (2014). The rumen fluid (pH 6.15) was collected from three sheep before morning feeding. The sheep were provided with ad libitum access to grass hay and supplemented with 300 g of concentrate per day. Incubations were done over 0, 2, 4, 6, 10, 24, 48 and 72 h. The 0‐h incubation was used for determination of the soluble fraction. At the end of each incubation period, the flasks were immersed in an ice bath to stop bacterial activity, the nylon bags were removed from the flasks and rinsed and agitated thrice in a beaker with tap water and a magnetic stirrer (for 10 min each time) and then dried in a forced air oven for 48 h at 50°C. Dry material was used to determine the degradation of DM, CP using 50 mg of the remaining sample (micro‐Kjeldahl; AOAC 1990) and amino acid profile.

Subsequently, an in vitro incubation was performed to determine the small intestine digestibility of the rumen undegraded crude protein according to the protocol described by Lima‐Orozco et al. (2014). About 150 mg of dried undegraded residues from the 10 h rumen incubation was placed into a new nylon bag, sealed and incubated in an Erlenmeyer flask containing hydrochloric acid (HCl)‐pepsin solution for 2 h at 39°C in a shaking incubator. Thereafter, nylon bags were washed thrice 10 min with distilled water and placed in the flask containing pancreatin solution and incubated for 2 h at 39°C in a shaking incubator. Afterwards, an additional amount of 20 mL of phosphate buffer was added to the incubation flask which was kept overnight at 39°C in the incubator without shaking. Finally, the nylon bags were washed three times 10 min with distilled water and placed in the oven to dry at 50°C for 24 h.

To evaluate the fermentability of washable fractions from atella samples, an assessment was conducted based on their volatile fatty acid (VFA) production. Initially, 500 mg of dry atella sample was placed in nylon bags and washed thrice in 150 mL of water in a beaker using a magnetic stirrer for 10 min per wash. The wash liquids collected from each washing cycle were pooled together and subjected to freeze‐drying. Subsequently, the resulting freeze‐dried washable fractions were incubated for 10 h in an in vitro rumen simulation to determine VFA production.

The total phenol content in the original atella samples and the 0‐h residues post‐washing with water was quantified following the procedures of Dewanto et al. (2002). Total phenol content in the washable fraction was calculated based on the differences between pre‐ and post‐wash values.

### 2.4 Calculations

Disappearance data of DM and CP were fitted to the exponential model developed by Ørskov and McDonald (1979). Effective rumen degradability (ERD, g/g) of DM and CP was calculated using a hypothetical fractional rate of passage of the particulate matter (k_p_) in the rumen of 0.03/h (Ørskov and McDonald 1979). The equations for disappearance and ERD of DM and CP were described by Tadesse et al. (2022).

The overall in vitro DM and CP digestibility (TIVDMD, g/g DM; TIVCPD, g/g CP) content was computed as the sum of the rumen degradable DM or CP and the amount of rumen undegradable DM or CP which was digested post‐ruminally (stomach and the small intestine simulation).

Gas chromatography was used to quantify VFA at the end of each incubation period in rumen fluid samples taken from each flask as described by Gadeyne et al. (2016).

### 2.5 Statistical Analysis

For normally distributed and square root transformed parameters, the mixed model following restricted maximum likelihood procedure was performed using JMP version16 statistical software.

$$Y_{\text{ijkl}}=\mu +{\mathrm{HTr}}_{i=1-2}+{\mathrm{CT}}_{j=1-2}+{\mathrm{HTr}}_{i}\times {\mathrm{CT}}_{j}+B_{k}+\varepsilon_{\text{ijkl}},$$

where *Y*_*ijkl*_ = dependent factors (Ruminal degradability and intestinal digestibility of DM, CP and amino acids etc.); HTr_*i* =1–2_ = heat treatment (high temperature vs. low temperature); CT_*j*=1–2_ = cereal type (maize vs. sorghum); HTr_*i*_ × CT_*j*_ = interaction between heat treatment and cereal type; *B*_*k*_ = the random effect of the tella (local beer) maker, and *ε*_*ijkl*_ = residual error. Least square mean differences were determined by a Tukey test with significance declared at *p* < 0.05. Tendencies were declared at 0.05 ≤ *p* < 0.10. Linear regression equations were developed between and intestinal digestibility of bypass crude protein with acid detergent insoluble CP, and with a (redness value).

## 3 Results

### 3.1 Rumen Degradability and Intestinal Digestibility of Dry Matter and Crude Protein

As observed for DM degradability (Table 1, *p* < 0.001), CP from maize‐derived atella was degraded less compared with CP from sorghum‐derived CP (Table 1, *p* < 0.001). This difference was mainly related to a considerably higher proportion of soluble CP (Table 1, *p* < 0.001) in sorghum‐derived CP as the potentially degradable fraction (Table 1, *p* = 0.713) did not differ between both cereal types. Temperature‐related differences particularly originated postruminally as effectively rumen degradable protein was not affected by the heat treatment (Table 1, *p* = 0.294), in line with the absence of a heat‐related effect for overall DM degradability (Table 1, *p* = 0.930).

**TABLE 1:** In vitro ruminal degradation characteristics (0–72 h) and intestinal digestibility of dry matter (DM) and crude protein (CP) of atella from tella prepared at two baking temperatures and from two cereal grain types. Least square means and standard error of the mean (SEM) are presented.

Parameters	Treatments				SEM	*p*		
	Maize		Sorghum			CT	HTr	CT × HTr
	LT	HT	LT	HT				
*DM (g/g DM, unless stated otherwise)*								
*a*	0.274	0.312	0.388	0.406	0.0214	< 0.001	0.033	0.392
*b* ^1^	0.546	0.442	0.360	0.338	[0.365, 0.473]^2^	< 0.001	0.027	0.149
*k*_*f*_ (/h)	0.037	0.042	0.051	0.051	0.0073	0.002	0.361	0.404
ERDDM	0.563	0.562	0.610	0.612	0.0185	< 0.001	0.930	0.726
TIVDMD	0.836^a^	0.794^b^	0.783^bc^	0.771^c^	0.0146	< 0.001	< 0.001	0.005
*CP (g/g CP, unless stated otherwise)*								
CP (g/kg DM)	182	168	191	184	13.1	< 0.001	< 0.001	0.060
ADICP	0.168	0.250	0.356	0.374	0.0212	< 0.001	0.035	0.152
*a*	0.084	0.093	0.184	0.244	0.0195	< 0.001	0.123	0.242
*b*	0.440	0.360	0.341	0.305	0.0928	0.713	0.270	0.562
*k*_*f*_ (/h)	0.071	0.103	0.130	0.081	0.0327	0.418	0.698	0.094
ERDCP	0.316	0.334	0.442	0.452	0.0218	< 0.001	0.294	0.752
RUP	0.684	0.666	0.558	0.548	0.0218	< 0.001	0.294	0.752
RDP/RDDM (g/kg)^1^	102	100	139	136	[102, 142]^2^	< 0.001	0.620	0.979
dBPCP (g/g BPCP)	0.907	0.807	0.715	0.649	0.0114	< 0.001	< 0.001	0.188
dBPCP	0.620	0.538	0.400	0.356	0.0195	< 0.001	0.002	0.204
TIVCPD	0.936^a^	0.872^b^	0.842^c^	0.808^d^	0.0076	< 0.001	< 0.001	0.037

*Note:* ^a,b,c,d^Means in a row with different superscripts are significantly different when annotated with different letters (*p* < 0.05).

Abbreviations: *a*, soluble fraction; ADICP, acid detergent insoluble crude protein; *b*, potentially rumen degradable fraction; BPCP, bypass crude protein; CT, cereal type; dBPCP, post‐ruminally digestible bypass crude protein; ERDCP, effective rumen degradability of crude protein; ERDDM, effective rumen degradability of dry matter; HT, high‐temperature heat treatment; HTr, heat treatment; *k*_*f*_, fractional degradation rate in the rumen; LT, low‐temperature heat treatment; RDDM, rumen degradable dry matter; RDP, rumen degradable protein; RUP, rumen undegradable protein; TIVCPD, total in vitro crude protein digestibility; TIVDMD, total in vitro dry matter digestibility.

^1^ Back transformed least square means are presented for the square root transformed values.

^2^ 95% confidence interval values.

Interaction effects between cereal type and heat treatment were observed for total in vitro DM digestibility (TIVDMD; Table 1, *p*_CT×HTr_ = 0.005) and total in vitro CP digestibility (TIVCPD; Table 1, *p*_CT×HTr_ = 0.037). In maize‐derived atella, increased heating temperature reduced TIVDMD and TIVCPD by 0.042 g/g DM and 0.064 g/g CP, respectively, whereas the reductions were less pronounced in sorghum‐derived atella.

### 3.2 Rumen Degradability and Intestinal Digestibility of Amino Acid

The AA profile of atella from tella prepared from the two cereal grain types and at either of the two baking temperatures is presented in Table 2. Rumen degradation of individual amino acids after 10 h of in vitro incubation varied between cereal types (*p* < 0.05), except tryptophan (Table 3). In maize‐derived atella, the rumen degradability of individual AA ranged from 0.204 to 0.361 g/g, with sulfur‐containing AA and lysine being most degraded, and tryptophan the least. In sorghum‐derived atella, the rumen degradation of individual AA ranged from 0.190 to 0.440 g/g, with sulfur AA, histidine, and lysine being most degraded, and tryptophan was the most resistant to ruminal microbial degradation. Except for valine, heat treatment did not influence rumen degradation of individual AA after 10 h of incubation.

**TABLE 2:** Amino acid (AA) composition (g/kg CP) of atella from tella prepared at two baking temperatures and from two cereal grain types.

Amino acid (g/kg CP)	Treatments			
	Maize		Sorghum	
	LT	HT	LT	HT
Alanine	68.4	60.4	76.5	72.3
Arginine	46.2	37.4	44.7	40.6
Aspartate*	68.3	61.0	69.3	66.3
Glutamate**	168	151	179	168
Glycine	37.7	35.3	37.9	37.1
Histidine	24.8	22.5	21.9	21.4
Isoleucine	39.1	34.2	41.6	39.3
Leucine	114	104	112	106
Lysine	28.7	23.5	26.0	23.6
Sulfur AA***	48.1	43.6	44.6	41.5
Phenylalanine	50.3	46.5	50.4	47.8
Serine	49.8	42.3	46.4	42.8
Threonine	38.5	33.7	36.3	34.2
Tryptophane	7.43	6.55	9.20	9.18
Tyrosine	39.6	36.9	39.2	37.1
Valine	45.1	44.9	46.4	44.1

Abbreviations: HT, high‐temperature heat treatment; LT, low‐temperature heat treatment.

* Sum of asparagine and aspartate.

** Sum of glutamine and glutamate.

*** Sulfur AA, sulfur‐containing amino acids (methionine + cysteine).

**TABLE 3:** In vitro ruminal degradation of amino acid (AA) and total CP after 10 h of ruminal incubation of atella from tella prepared at two baking temperatures and from two cereal grain types. Least square means and standard error of the mean (SEM) are presented.

Amino acid (g/g AA)	Treatment				SEM	*p*		
	Maize		Sorghum					
	LT	HT	LT	HT		CT	HTr	CT × HTr
Alanine	0.226	0.231	0.388	0.381	0.0518	< 0.001	0.930	0.703
Arginine	0.293	0.271	0.416	0.404	0.0627	< 0.001	0.284	0.722
Aspartate*	0.276	0.264	0.405	0.397	0.0574	< 0.001	0.535	0.921
Glutamate**	0.246	0.258	0.402	0.398	0.0424	< 0.001	0.702	0.444
Glycine	0.267	0.272	0.396	0.396	0.0519	< 0.001	0.843	0.846
Histidine	0.260	0.273	0.411	0.434	0.0463	< 0.001	0.170	0.686
Isoleucine	0.275	0.250	0.417	0.407	0.0599	< 0.001	0.239	0.608
Leucine	0.220	0.254	0.392	0.385	0.0480	< 0.001	0.257	0.104
Lysine	0.311	0.309	0.417	0.418	0.0718	< 0.001	0.962	0.924
Phenylanine	0.230	0.256	0.387	0.380	0.0500	< 0.001	0.464	0.187
Serine	0.258	0.259	0.405	0.397	0.0539	< 0.001	0.823	0.732
Sulfur AA***	0.428^c^	0.338^d^	0.522^a^	0.477^b^	0.0245	< 0.001	< 0.001	0.038
Threonine	0.274	0.275	0.410	0.409	0.0566	< 0.001	0.998	0.940
Tryptophan	0.146	0.261	0.178	0.201	0.1171	0.838	0.347	0.524
Tyrosine	0.239	0.272	0.393	0.382	0.0520	< 0.001	0.383	0.106
Valine	0.254^c^	0.316^b^	0.400^a^	0.388^a^	0.0511	< 0.001	0.030	0.004
CP (g/g CP)	0.220	0.232	0.381	0.394	0.0289	< 0.001	0.503	0.977

*Note:* ^a,b,c,d^Means in a row with different superscripts are significantly different when annotated with different letters (*p* < 0.05).

Abbreviations: CP, crude protein; CT, cereal type; HT, high‐temperature heat treatment; HTr, heat treatment; LT, low‐temperature heat treatment.

* Sum of asparagine and aspartate.

** Sum of glutamine and glutamate.

*** Sulfur AA, sulfur‐containing amino acids (methionine + cysteine).

The CP degradation of maize‐ and sorghum‐derived atella after 10 h rumen incubation was 0.226 and 0.388 g/g, respectively (Table 3). For maize‐derived atella, amino acids exhibited similar degradability to CP (relative differences −2.3% to +5.9%), except lysine (+8.5%) and sulfur AA (+15.7%), which were higher. In sorghum‐derived atella, all AA, except tryptophan (−19.8%) and sulfur AA (+11.2%), showed similar degradation to CP (relative differences −0.4% to +3.5%). Intestinal digestibility of the rumen bypass fraction of all AA was affected (*p* < 0.05) by cereal type or heat treatment (Table 4). Interaction effects (*p* < 0.05) between cereal type and heat treatment were observed for the intestinal digestibility of arginine, aspartate, glycine, histidine, lysine, threonine and valine, with a tendency for isoleucine (*p* = 0.050) (Table 4). Increasing the heating temperature reduced the digestibility of these amino acids in both cereal types, with the reduction being more pronounced in maize‐derived atella (7.0% for lysine to 10.5% for glycine) compared to sorghum‐derived atella (3.6% for lysine to 6.6% for glycine).

**TABLE 4:** In vitro intestinal digestibility of rumen undegraded amino acid of atella from tella prepared at two baking temperatures and from two cereal grain types. Least square means and standard error of the mean (SEM) are presented.

Amino acid (g/g AA)	Treatment				SEM	*p*		
	Maize		Sorghum					
	LT	HT	LT	HT		CT	HTr	CT × HTr
Alanine	0.941	0.871	0.656	0.612	0.0118	< 0.001	< 0.001	0.182
Arginine	0.909^a^	0.839^b^	0.727^c^	0.690^d^	0.0087	< 0.001	< 0.001	0.015
Aspartate*	0.931^a^	0.850^b^	0.708^c^	0.664^d^	0.0082	< 0.001	< 0.001	0.012
Glutamate**	0.935	0.876	0.665	0.621	0.0133	< 0.001	0.001	0.456
Glycine	0.879^a^	0.787^b^	0.653^c^	0.610^d^	0.0090	< 0.001	< 0.001	0.002
Histidine	0.863^a^	0.784^b^	0.639^c^	0.600^d^	0.0121	< 0.001	< 0.001	0.034
Isoleucine	0.930^A^	0.851^B^	0.686^C^	0.640^D^	0.0107	< 0.001	< 0.001	0.050
Leucine	0.944	0.886	0.654	0.611	0.0127	< 0.001	< 0.001	0.461
Lysine	0.901^a^	0.838^b^	0.777^c^	0.749^d^	0.0092	< 0.001	< 0.001	0.016
Phenylanine	0.945	0.877	0.690	0.646	0.0106	< 0.001	< 0.001	0.176
Proline	0.883	0.816	0.606	0.560	0.0151	< 0.001	< 0.001	0.321
Serine	0.924	0.865	0.678	0.639	0.0104	< 0.001	0.001	0.193
Sulfur AA***	0.758	0.667	0.490	0.417	0.0235	< 0.001	< 0.001	0.378
Threonine	0.895^a^	0.823^b^	0.689^c^	0.650^d^	0.0091	< 0.001	< 0.001	0.023
Tryptophan	0.864	0.796	0.644	0.621	0.0229	< 0.001	0.058	0.309
Tyrosine	0.943	0.879	0.690	0.649	0.0101	< 0.001	< 0.001	0.185
Valine	0.911^a^	0.825^b^	0.688^c^	0.639^d^	0.0103	< 0.001	< 0.001	0.024

*Note:* ^a,b,c,d^Means in a row with different superscripts are significantly different when annotated by small letters (*p* < 0.05), while tendencies are denoted by capital letters (0.05 ≤ *p* < 0.10).

Abbreviations: CT, cereal type; HT, high‐temperature heat treatment; HTr, heat treatment; LT, low‐temperature heat treatment.

* Sum of asparagine and aspartate.

** Sum of glutamine and glutamate.

*** Sulfur AA = sulfur‐containing amino acids (methionine + cysteine).

The first four limiting essential amino acids (EAA) in maize‐ and sorghum‐derived atella were lysine, methionine, isoleucine, and valine (Table 5). The ratios of the proportions of these EAA in digestible bypass amino acid (dBPAA) of atella relative to EAA in milk protein in maize‐derived atella was between 19.5% to 42.5%, while in sorghum‐derived atella ranged between 13.5% to 27.5%.

**TABLE 5:** Essential amino acid (EAA, g/16 g N) profile in milk and EAA ratios (% milk EAA) for post‐ruminally digestible bypass amino acid (dBPAA, g/16 g N) of atella from tella prepared at two baking temperatures and from two cereal grain types as an index of protein quality.

Essential AA	Milk^a^	Treatment				Treatment			
		Maize		Sorghum		Maize		Sorghum	
		LT	HT	LT	HT	LT	HT	LT	HT
	(g/16 g N)					(% relative to milk)^b^			
Lysine	8.1	1.78	1.36	1.17	1.03	22	17	14	13
Methionine	2.6	0.93	0.85	0.52	0.45	36	33	20	17.3
Isoleucine	5.9	2.63	2.18	1.66	1.49	45	37	28	25
Valine	6.6	3.07	2.53	1.91	1.72	47	38	29	26
Threonine	4.6	2.50	2.01	1.47	1.31	54	44	32	29
Histidine	2.7	1.58	1.28	0.82	0.73	59	47	30	27
Phenylanine	4.9	3.66	3.03	2.13	1.91	75	62	44	39
Arginine	3.5	2.97	2.29	1.90	1.67	85	65	54	48
Leucine	9.7	8.35	6.82	4.43	3.96	86	70	46	41

Abbreviations: HT, high‐temperature heat treatment; LT, low‐temperature heat treatment.

^a^ EAA profile for milk calculated from the data reported by Spires et al. (1975).

^b^ Percentage of EAA in atella relative to that of milk.

### 3.3 Color Measurements

The color measurements showed an interaction effect between cereal type and heat treatment (*p* < 0.05), affecting the *L* and *a* values (Table 6). Maize and sorghum flour (raw material) used for the preparation of tella had *L* (lightness) values of 84.6 and 70.6, respectively (data not shown), being 2.2 and 1.74 times lighter than their corresponding atella samples. Low‐temperature‐treated atella was lighter (+6.8, *p* < 0.001) than the high‐temperature‐treated atella.

**TABLE 6:** Color score of atella from tella prepared at two baking temperatures and from two cereal grain types. Least square means and standard error of the mean (SEM) are presented.

Hunter Lab color score	Treatments				SEM	*p*		
	Maize		Sorghum			CT	HTr	CT × HTr
	LT	HT	LT	HT				
*L*	41.8^a^	33.8^c^	42.5^a^	37.8^b^	1.00	0.005	< 0.001	0.032
*a*	2.03^c^	3.04^b^	4.60^a^	4.52^a^	0.132	< 0.001	0.007	0.003
*b*	16.5	12.2	16.4	14.2	0.74	0.130	< 0.0001	0.081

*Note:* ^a,b,c^Means in a row with different superscripts are significantly different when annotated by small letters (*p* < 0.05).

Abbreviations: CT, cereal type; HT, high‐temperature heat treatment; HTr, heat treatment; *L*, lightness of sample, 0 = black, 100 = white; the greater the value of *a* and *b*, the greater degree of redness and yellowness, respectively; LT, low‐temperature heat treatment.

## 4 Discussion

### 4.1 Rumen Degradability

It was expected that the increase in baking temperature of the pancake would decrease overall and particularly protein degradability in the rumen (McKinnon et al. 1995; Dakowski et al. 1996). However, no difference was observed in the effective rumen degradability of dry matter and protein for the atella samples treated at high vs. low temperatures. In agreement to the present findings, several studies [Lykos and Varga 1995 for raw vs. roasted (144°C) grounded soybean; McNiven et al. 1994 for roasted corn grain at 118°C vs. 163°C and Armentano et al. 1986 for wet brewers grain oven dried at 50°C vs. 150°C] reported no difference in the effective rumen DM and CP degradability with the increase of heating temperature.

Regarding differences in effective rumen degradability of DM and CP between cereal types, sorghum‐derived atella exhibited higher degradability than maize‐derived atella, attributed to its higher amount of soluble fraction. Consistent with this, previous studies have shown sorghum to have a higher soluble CP fraction compared to maize (23.4% vs. 16.9%: Erasmus et al. 1990; 41% vs. 9.7%: Heuzé et al. 2015, 2017). Despite the higher ruminal degradation of dry matter for sorghum‐derived atella (Table 1 and Figure S1), this did not correspond to a proportional increase in total volatile fatty acid production (Figure S2), possibly due to the presence of notable unfermented washable fractions. To evaluate the fermentability of washable fractions from maize‐ vs. sorghum‐derived atella, a 10‐h in vitro rumen incubation was performed. The 10‐h total volatile fatty acid produced from freeze‐dried washable fractions from maize and sorghum‐ derived atella were 4.21 and 3.83 μmol/mg washable DM, which is indicative of lower fermentability of the washable fraction from sorghum‐derived atella. Phenolic compounds, particularly tannins, impair ruminal carbohydrate fermentation through multiple mechanisms: (i) inhibition of microbial enzymes such as amylases and cellulases, reducing starch and fiber degradation; (ii) suppression of amylolytic and cellulolytic bacterial activity, lowering fermentation efficiency; and (iii) formation of tannin–protein complexes, which reduce protein fermentation and limit nitrogen availability required for optimal microbial fermentation (Amanzougarene et al. 2018). Additionally, total phenols content in the washable fractions was 1.27 mg/g for the maize‐derived atella and 1.45 mg/g for the sorghum‐derived atella, suggesting that the higher phenol content in the washable fraction of the sorghum‐derived atella may have contributed to its reduced fermentability.

The effective rumen degradability values for DM and CP in maize‐derived atella were consistent with those reported by Tadesse et al. (2022). While atella has been identified as an interesting source of rumen undegradable protein (RUP) in small‐scale dairy farms (Tadesse et al. 2022), relying on atella as the main protein source fails to meet the minimum threshold of 125 g RDP/kg rumen degradable dry matter (RDDM) for optimal ruminal microbial yield (Tamminga et al. 1990; Ibrahim et al. 1995). From a practical standpoint, wheat bran, with 144–207 g RDP/kg RDDM, is commonly used in dairy farms as a supplement that could supply extra energy while also complementing rumen degradable protein shortages for optimal rumen microbial yield (Tadesse et al. 2025).

Patterns of rumen degradation of CP and mean AA were generally comparable to those reported for other protein sources, including brewer's grain, soybean meal, and fish meal, with relative differences between CP and mean AA degradation typically ranging from −4.0% to +4.0% (Susmel et al. 1989). In the present study, degradation of individual AA in maize‐derived atella was similar to that observed for brewer's grain, with lysine (+10%), methionine (+14%), and other AA ranging from −4.0% to +8%. Sulfur‐containing AA and lysine were the most degradable AA in both maize‐ and sorghum‐derived atella. Lysine has consistently been reported as highly degradable in the rumen across diverse feeds, including corn and sorghum (Erasmus et al. 1994), hemp seed cake (Karlsson et al. 2012), and dried brewers' grain and soybean meal (Susmel et al. 1989).

However, methionine degradation exhibited inconsistency, reported as highly rumen degradable in hemp seed cake (Karlsson et al. 2012) and dried brewers' grain (Susmel et al. 1989) but resistant in corn and sorghum (Erasmus et al. 1994), sunflower meal (Molina Alcaide et al. 2003), and extruded white lupins (Cros et al. 1992). In the current study, individual quantification of cysteine and methionine was not conducted. Instead, the combined concentration of sulfur‐containing amino acids (cysteine + methionine) was estimated from the sulfur content of the sample, which should be considered when interpreting current results.

Tryptophan exhibited the greatest resistance to ruminal degradation among essential AA in both maize‐ and sorghum‐derived atella. This finding contrasts with previous reports identifying tryptophan as the most rumen degradable essential AA in cottonseed meal, sunflower seed meal, and distillers dried grains with solubles (Gao et al. 2015). The lower degradability of tryptophan observed in this experiment is difficult to explain. Additionally, literature data on the ruminal degradation of tryptophan are limited, likely due to analytical challenges. The variation in the ruminal degradation of individual amino acids could be linked to feed characteristics (Crooker et al. 1987; Erasmus et al. 1994) and differences in the preferential degradation order of the AA by the rumen microbes (Tamminga 1979; Gao et al. 2015).

### 4.2 Small Intestine Digestibility

**FIGURE 1:**
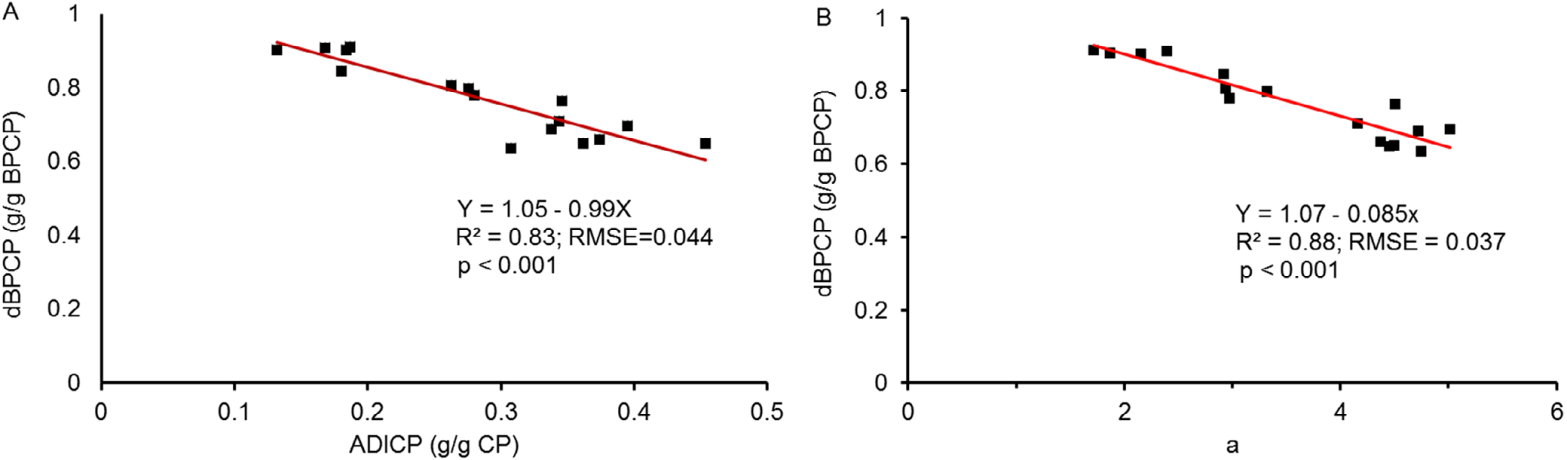
The association between digestible bypass crude protein (dBPCP, g/g BPCP) with acid detergent insoluble crude protein (ADICP, g/g CP) (A) and with a (redness value) (B).

The CP intestinal digestibility of the 10 h rumen incubation residues revealed that low‐ temperature‐treated atella exhibited higher digestible CP than high‐temperature‐treated atella, both on bypass and feed CP basis. As expected, the rise of baking temperature of the pancake reduced intestinal digestibility of bypass protein and the total in vitro digestible crude protein. In support of the current findings, the increase of heating temperature differences of about 20°C between 125°C–130°C to 145°C–150°C for 30 to 60 min for canola meals (McKinnon et al. 1995) and soybean or cotton seed cakes (Schroeder et al. 1995) resulted in a decrease in intestinal digestibility of bypass protein (−35% to −39%) and total in vitro digestible crude protein (−22.5% to −31%) and an increase in acid detergent insoluble crude protein (ADICP) (21.8% to 31.9%). ADICP has been identified before serving as a good indicator for assessing unavailable protein (Van Soest and Mason 1991; Licitra et al. 1996). Elevated temperatures and extended heating durations have been observed to increase ADICP contents via Maillard reactions between sugars and amino acids (Moshtaghi Nia and Ingalls 1995) or crosslinking between lignin and nitrogenous compounds (Van Soest and Mason 1991; Licitra et al. 1996). In the present findings, a strong negative association (*R* ^2^ = 0.83; *p* < 0.001; Figure 1A) was observed between ADICP and the intestinal digestibility of bypass protein, consistent with findings by Schroeder et al. (1995) concerning soybean oilcake (*R* ^2^ = 0.86). As such, the simple ADICP measure could serve as a proxy for the digestibility measure, which is more complicated and expensive to determine. The physical appearance, for example, color, could be another easy proxy particularly when variation in digestibility measures are provoked by temperature treatments. The pancakes baked at low or high temperature differed considerably with the latter being black colored. As such, a high negative correlation (*R* ^2^ = 0.88; *p* < 0.001) between the intestinal digestibility of bypass protein and redness value was observed (Figure 1B). The assessment of the amino acid profile is paramount in delineating the quality of ruminant feeds with high levels of rumen‐undegradable protein (Dakowski et al. 1996). The increase in baking temperature from 109°C to 159°C of the pancake resulted in a decrease of intestinal digestibility of individual AA in maize‐derived atella (from −6.3% to −9.2%) or sorghum‐derived atella (from −2.8% to −4.9%). In support of our findings, the increase of the heating temperature of rapeseed meal from 130°C to 150°C led to a reduction in the intestinal digestibility of individual amino acids of rumen undegraded residue, with a range of decrease between 6.6% and 16.6% (Dakowski et al. 1996). Sulfur‐containing AA exhibited lower intestinal digestibility compared to other AA, potentially due to stable disulfide bonds between cysteine molecules, which resist enzymatic degradation (Hamaker et al. 1987; Fombang 2005). Increased baking temperatures further reduce S‐containing AA digestibility, likely due to enhanced disulfide linkage formation (Hamaker et al. 1987). Furthermore, a considerable portion of sulfur‐AA is degraded in the rumen, leaving an undegraded fraction that may contain more indigestible components.

The intestinal protein or AA digestibility of rumen undegraded residues is also influenced by the fiber content of the feed (Čerešňáková et al. 2002) and protein cross‐linking (Duodu et al. 2003). In the present findings, the sorghum‐derived atella had lower bypass intestinal digestibility of CP or AA, which is attributed to the higher concentration of ADF (245 g/kg DM) and possibly the presence of more crosslinking of γ‐ and β‐kafirin proteins (Duodu et al. 2003).

Lysine and Methionine are the primary limiting amino acids for milk protein production in dairy cows (NRC 2001; Schwab and Whitehouse 2022), a finding corroborated by our experiment (Table 5). The amount of rumen undegradable protein and its amino acid composition could influence this limitation (Schwab and Whitehouse 2022). Maize grain is typically deficient in lysine, methionine + cysteine, and tryptophan (Liu et al. 2021). Although supplementing rumen‐protected lysine and methionine can be considered to enhance post‐ruminal amino acid supply (Elsaadawy et al. 2022), such products are more relevant as a fine‐tuning strategy in highly specialized systems with precise diet formulation and monitoring rather than a priority approach for smallholder systems in Africa. In the latter context, the main focus should be on optimizing microbial protein production by supplying adequate amounts of fermentable carbohydrates with rumen‐degradable protein (Varga 2007) and ensuring sufficient sulfur to support microbial synthesis of sulfur‐containing amino acids (da Silva et al. 2014).

## 5 Conclusions

Heat treatment did not affect rumen degradability of atella crude protein and 10‐h degradation of individual amino acids (AA), but notable variation was observed in intestinal digestibility of rumen undegradable protein and individual AA. The two cereal types exhibited differences in both ruminal degradability and intestinal digestibility of these parameters. Maize‐derived atella demonstrated greater rumen undegradable fractions and higher intestinal digestibility of rumen undegradable protein and individual AA levels as compared to the corresponding sorghum‐derived atella. A strong negative correlation existed between acid detergent insoluble crude protein and intestinal digestibility of bypass protein. Low‐temperature‐treated maize‐derived atella showed superior total in vitro crude protein digestibility and intestinal digestibility of individual AA (arginine, aspartate, glycine, histidine, isoleucine, lysine, threonine and valine) compared to other atella types. Atella from sorghum or maize was deficient in sulfur content and showed lesser digestion of sulfur‐containing AA in the small intestine compared to other AA. Lysine and methionine from maize‐ or sorghum‐derived atella were identified as the primary and secondary limiting amino acids for milk protein production in dairy cows. Given that the relatively high proportions of rumen undegradable protein in cereal‐based atella diets should be balanced with feed ingredients high in rumen‐degradable protein.

## Author Contributions

**Alemayehu Tadesse:** conceptualization (equal), data curation (lead), formal analysis (lead), funding acquisition (supporting), investigation (equal), methodology (equal), writing – original draft (lead), writing – review and editing (equal). **Nympha De Neve:** investigation (supporting), writing – review and editing (supporting). **Yayneshet Tesfay:** investigation (supporting), writing – review and editing (supporting). **Pieter Vermeir:** investigation (supporting), resources (supporting), writing – review and editing (supporting). **Bruno De Meulenaer:** investigation (supporting), resources (supporting), writing – review and editing (supporting). **Veerle Fievez:** conceptualization (lead), formal analysis (supporting), funding acquisition (lead), investigation (lead), methodology (lead), resources (lead), supervision (lead), writing – original draft (supporting), writing – review and editing (lead).

## Funding

This work was supported by Bijzonder Onderzoeksfonds UGent.

## Disclosure

Financial support statement: The PhD scholarship to Alemayehu Tadesse, provided by the Special Research Fund (BOF) of Ghent University, is gratefully acknowledged. Our appreciation also extends to BOF special research fund for the extension of the PhD trajectory that was delayed due to COVID‐19.

## Ethics Statement

The ethical commission of the Institute for Agricultural and Fisheries Research (ILVO, Belgium; file number EC2015‐257) approved the fistulation of the animals from which the rumen fluid was sampled.

## Conflicts of Interest

The authors declare no conflicts of interest.

## Supporting information


**Figure S1:** In vitro ruminal degradation characteristics of atella DM based on in vitro rumen incubation during various periods (0–72 h). Atella was derived from tella prepared at two baking temperatures and from two cereal grain types.
**Figure S2:** In vitro ruminal total volatile fatty acid (TVFA) produced from the incubated atella samples based on in vitro rumen incubation during various periods (0–72 h). Atella was derived from tella prepared at two baking temperatures and from two cereal grain types.

## Data Availability

The data that support the findings of this study are available from the corresponding author upon reasonable request.
