# Supplementary material for: Characterizing Origin of Differences in Chemical Composition, Rumen Degradability, Intestinal Digestibility and Amino Acid Profile of Traditional Beer Brewing Byproducts
Source: Food Sci Nutr. 2025 Dec 30;14(1):e71380. doi: 10.1002/fsn3.71380 (PMC12750511; doi:10.1002/fsn3.71380)
Supplement: Supplementary file 1 [file FSN3-14-e71380-s001.odt]

## Supplementary figures

A graph of a curve

Description automatically generated with medium confidenceSupplementary Figure 1 In vitro ruminal degradation characteristics of atella DM based on in vitro rumen incubation during various periods (0–72h). Atella was derived from tella prepared at two baking temperatures and from two cereal grain types. 

Abbreviations: HTMA, high-temperature heat-treated maize-derived atella; LTMA, low-temperature heat-treated maize-derived atella; HTSA, high-temperature heat-treated sorghum-derived atella; LTSA, low-temperature heat-treated sorghum-derived atella.

A graph of a curve

Description automatically generated with medium confidence

Supplementary Figure 2 In vitro ruminal total volatile fatty acid (TVFA) produced from the incubated atella samples based on in vitro rumen incubation during various periods (0–72h). Atella was derived from tella prepared at two baking temperatures and from two cereal grain types.

 Abbreviations: HTMA, high-temperature heat-treated maize-derived atella; LTMA, low-temperature heat-treated maize-derived atella; HTSA, high-temperature heat-treated sorghum-derived atella; LTSA,
